# Effect of *IKZF1* deletions on signal transduction pathways in Philadelphia chromosome negative pediatric B-cell precursor acute lymphoblastic leukemia (BCP-ALL)

**DOI:** 10.1186/s40164-015-0017-y

**Published:** 2015-08-12

**Authors:** Naomi E van der Sligte, Frank J G Scherpen, Arja ter Elst, Victor Guryev, Frank N van Leeuwen, Eveline S J M de Bont

**Affiliations:** Division of Pediatric Oncology/Hematology, Department of Pediatrics, Beatrix Children’s Hospital, University Medical Center Groningen, University of Groningen, PO Box 30.001, 9700 RB Groningen, The Netherlands; European Research Institute for the Biology of Ageing, University Medical Center Groningen, University of Groningen, Groningen, The Netherlands; Laboratory of Pediatric Oncology, Department of Pediatrics, Radboud Institute for Molecular Life Sciences, Radboud University Medical Center, Nijmegen, The Netherlands

**Keywords:** Acute lymphoblastic leukemia, *IKZF1*, Signaling, Kinome profiling

## Abstract

**Background:**

*IKZF1* deletions are an unfavorable prognostic factor in children with Philadelphia chromosome positive (Ph^+^) as well as negative (Ph^−^) acute lymphoblastic leukemia (ALL). Although *IKZF1* deletions occur in 10–15% of Ph^−^ ALL cases, effects of *IKZF1* deletions on signaling pathways in this group have not been extensively studied. Therefore, in this study we aimed to study the effect of *IKZF1* deletions on active signal transduction pathways.

**Methods:**

Multiplex ligation-dependent probe amplification (MLPA) was used to determine *IKZF1* deletions and other copy number alterations in 109 pediatric B-Cell Precursor ALL (BCP-ALL) patients. Kinase activity profiling of 45 primary Ph^−^ BCP-ALL patients (31 *IKZF1* wild type patients and 14 patients harboring an *IKZF1* alteration) and western blot analysis of 14 pediatric BCP-ALL samples was performed to determine active signal transduction pathways.

**Results:**

Unsupervised hierarchical cluster analysis of kinome profiles of 45 pediatric Ph^−^ ALL cases showed no clustering based on *IKZF1* status. Comparing the phosphorylation intensities of peptides associated with signaling pathways known to be involved in BCP-ALL maintenance, we did not observe differences between the two groups. Western blot analysis of 14 pediatric BCP-ALL samples showed large variations in phosphorylation levels between the different ALL samples, independent of *IKZF1* status.

**Conclusions:**

Based on these results we conclude that, although *IKZF1* deletions appear to be an important clinical prognostic factor, we were unable to identify a unique *IKZF1* dependent protein expression signature in pediatric Ph^−^ ALL and consequently no specific targets for future therapy of Ph^−^*IKZF1* deleted BCP-ALL could be identified.

**Electronic supplementary material:**

The online version of this article (doi:10.1186/s40164-015-0017-y) contains supplementary material, which is available to authorized users.

## Background

Overall survival rates for children with Acute Lymphoblastic Leukemia (ALL), the most common type of cancer in children, are approaching 90% [1]. Historically, risk stratification of newly diagnosed children was based on age and white blood cell count (WBC), but nowadays includes extensive cytogenetic and molecular analyses. In the past 5 years, genome wide approaches, studying DNA copy number alterations in ALL, have identified novel molecular markers that can be used for further risk stratification, including *IKZF1* deletions as a predictor of poor outcome. *IKZF1* deletions can be identified in approximately 70% of the children with Philadelphia chromosome positive (Ph^+^) ALL and in 10–15% of the children with Philadelphia chromosome negative (Ph^−^) ALL and are associated with an increased relapse risk and decreased overall survival in both groups [2–4]. More recent studies indicate that the genomic context in which *IKZF1* deletions occur is more important for prognosis as for example *CRLF2* and *JAK2* mutations are more common in *IKZF1* deleted BCP-ALL [5–7]. In pediatric B-cell progenitor ALL (BCP-ALL), 80% of the *IKZF1* deletions are found in a Philadelphia chromosome negative background.

*IKZF1*, which encodes the transcription factor Ikaros, is essential for normal lymphoid development, whereas for erythroid and myeloid lineage differentiation *IKZF1* is less critical [8]. Mice deficient for *IKZF1* show a complete arrest in B-lymphocyte development while mice heterozygous for a dominant-negative mutation of *IKZF1* develop T cell leukemia and lymphoma with a 100% penetrance [9, 10]. During normal development, Ikaros restricts the G_1_-S transition of the cell cycle when it binds to the DNA, by regulating transcription of cell cycle regulator genes e.g. a positive effect on cell cycle inhibitors *CDKN1A* (p21Cip1) and *CDKN1B* (p27Kip1) [11]. Phosphorylation of Ikaros by casein kinase II (CK2) temporarily reduces Ikaros binding to DNA and thereby facilitates progression through the S phase of the cell cycle [11]. Furthermore, Ikaros can be phosphorylated by spleen tyrosine kinase (SYK) and bruton’s tyrosine kinase (BTK) [12, 13]. These phosphorylation events are essential for nuclear localization, regulation of DNA binding activity, and an optimal transcriptional function of Ikaros [12, 13].

*IKZF1* deletions observed in BCP-ALL are typically mono-allelic, either resulting in a loss of function or the expression of a dominant-negative isoform [14]. The dominant-negative isoforms lack the DNA binding N-terminal zinc fingers, preventing DNA binding after dimerization with Ikaros [15]. As a result, the control of Ikaros on the G_1_-S transition is abolished leading to hyperproliferation and the development of leukemia [11].

Although the cure rates for children with BCP-ALL have improved substantially, the outcome after ALL relapse remains poor. Since *IKZF1* deletions increase the risk of relapse, new therapeutic options aiming to improve cure rates for this specific subtype of ALL are needed. We have previously shown that insight into active signal transduction pathways allows identification of interesting targets for future therapy [16–19]. At the level of signal transduction, Iacobucci et al. showed on western blot analysis a higher STAT5 phosphorylation in *IKZF1* deleted compared to *IKZF1* wild type adult BCP-ALL patients with unknown cytogenetic background [20]. However, this observation might also be associated with BCR-ABL1 activity as in adult BCP-ALL patients *IKZF1* deletions are more common in Ph^+^ ALL [21]. Additionally, Ikaros-reconstitution in two *IKZF1* deleted Philadelphia positive ALL patients resulted in an upregulation of the B-cell receptor (BCR) signaling pathway and a concomitant cell cycle arrest; showing that in Ph^+^ ALL pre-B cell receptor signaling suppresses proliferation through an Ikaros-mediated cell cycle arrest [22]. Although *IKZF1* deletions in children are most commonly found in a Philadelphia negative background, the effect of *IKZF1* deletions on signaling pathways in Philadelphia negative ALL have not been extensively studied. Therefore, in this study we aimed to study the effect of *IKZF1* deletions on active signal transduction pathways in Philadelphia negative pediatric BCP-ALL using kinome profiling.

## Methods

### Patients

Primary blood and bone marrow samples from newly diagnosed ALL patients were collected after getting written informed consent in accordance with the regulations and protocols of the medical ethics committee of the University Medical Center Groningen. Overall, we collected material of 109 Philadelphia negative BCP-ALL patients. Mononuclear cells were isolated by Lympho-prep (Nycomed, Zürich, Switzerland) density gradients and cryopreserved in liquid nitrogen until use. The cryopreserved leukemia cells were thawed rapidly at 37°C and diluted in a 6 ml volume of newborn calf serum, as described earlier [23].

### DNA isolation

Genomic DNA was extracted from mononuclear cells using the QIAamp DNA easy kit (Qiagen, Hilden, Germany) according to the manufacturer’s instructions. All isolated DNA was quantified by NanoDrop spectrophotometry (NanoDrop, Wilmington, DE, USA).

### Multiplex ligation-dependent probe amplification (MLPA)

Targeted copy number screening of eight selected loci was performed in the cohort by multiplex ligation-dependent probe amplification (MLPA) using the P335-B2 SALSA MLPA kit (MRC-Holland, Amsterdam, The Netherlands). The assay includes probes for each of the eight exons of the *IKZF1* gene and is able to detect deletions of the whole gene as well as all types of focal intragenic deletions. Selected exons of the genes *BTG1, CDKN2A/B, EBF1, ETV6, PAX5, RB1* and the PAR1 region (approx. 230 kbp downstream of *SHOX*, *CRLF2*, *CSF2RA* and *IL3RA*) are also covered. Probe mix and hybridization buffer (MRC-Holland) were added in equal amounts to 50 ng of genomic DNA followed by heat denaturation and overnight hybridization of the probes at 60°C. Subsequently, ligation was performed and the ligation products were amplified by PCR using a 6-FAM fluorophore-labeled primer set (MRC-Holland). The amplification products were quantified and identified by capillary electrophoresis on an ABI 3730 DNA analyzer (Applied Biosystems, Foster City, CA, USA).

*Data analysis*: Data were analyzed using Gene Mapper v.4.0 software (Applied Biosystems). Normalization of the data was carried out by dividing the peak area of each probe by the average peak area of the control probes. This normalized peak pattern was divided by the average peak pattern of all the samples in the same experiment. The resulting values were 1 for every wild-type peak, 0.5 for heterozygous deletions and 1.5 for heterozygous duplications.

### PepChip

Kinase activity profiles of 45 primary Ph^−^ BCP-ALL patients were determined using the PepChip™ Kinomics microarray system (Pepscan, Lelystad, the Netherlands) and performed as described previously [18, 19]. The microarray contain 1,024 peptides in triplicate (1,008 unique target peptides and 16 peptides used for production) derived from known phosphorylation sites from human protein sequences that can be phosphorylated by the kinases in the sample lysate. Per sample, 0.5 × 10^6^ cells were lysed in 100 μl of M-PER Mammalian Protein Extraction Buffer containing 1 μl Phosphatase Inhibitor and 1 μl Protease Inhibitor (Pierce, Rockford, IL, United States). Peptide array incubation mix was produced by adding 10 μl of filter-cleared activation mix onto 90 μl cell lysate. Peptide array incubation mix was loaded on the chip and incubated for 2 h at 37°C in a closed humid box at saturated humidity. Subsequently, the peptide array was washed and blow dried with compressed air or N_2_ and the chips were exposed to a phospho-storage screen for 24–96 h. The amount of bound ^33^P-labelled ATP to the peptides specifies the amount of peptide phosphorylation and was analyzed with array software (ScanAlyze, Eisen Lab, University of California at Berkely, Berkely, CA, United States).

*Data analysis*: Data were analyzed as described previously [18, 19]. In short, background was subtracted and the spot intensities were quantile normalized. A Pearson’s correlation coefficient was determined over the triplicates (Excel 2003, Microsoft Office, Redmond, WA, United States). Slides with a correlation <0.6 over the triplicates were excluded from further analysis. The correlation over the triplicates was <0.6 in none of the samples. Cluster, statistical and heatmap analysis were performed using Qlucore Omics Explorer 3.0 (Qlucor AB, Lund, Sweden). The file containing the processed raw data can be found in the additional information (Additional file 1: Table S1).

### Western blot analysis

Primary ALL cells were solved in laemmli sample buffer (Bio-Rad laboratories, Veenendaal, the Netherlands). Proteins were separated by sodium dodecyl sulphate–polyacrylamide gel electrophoresis, and transported to nitrocellulose membranes. Membranes were blocked in 7.5% skimmed milk and incubated overnight with primary antibodies for phospho-Src_Y416, phospho-Syk_Y323, phospho-ERK1/2_T202/Y204, phospho-CREB_S133, phospho-p38_T180/Y182, phospho-Akt_S473, phospho-mTOR_S2448, phospho-GSK3α/β_S21/S9, phospho-MDM2_S166, phospho-Chk2_T68, phospho-RB1_S807/811, phospho-STAT3_Y705, phospho-STAT5_Y694 (Cell Signaling, Danvers, MA, USA), or phospho-p27_T187 (Abgent, San Diego, CA, United States) and for 1 h with HRP conjugated secondary antibodies (Dako, Glosturp, Denmark). Protein bands were visualized using the ChemiDoc MP imaging system (Bio-Rad, Hercules, CA, United States) and ImageLab software (version 5.0, Bio-Rad Laboratories). Loading control was visualized using β-actin (Santa Cruz Biotechnology, Dallas, TX, United States).

## Results

### Generation of kinase activity profiles in *IKZF1* deleted versus *IKZF1* wild type Philadelphia negative pediatric BCP-ALL

**Fig. 1:**
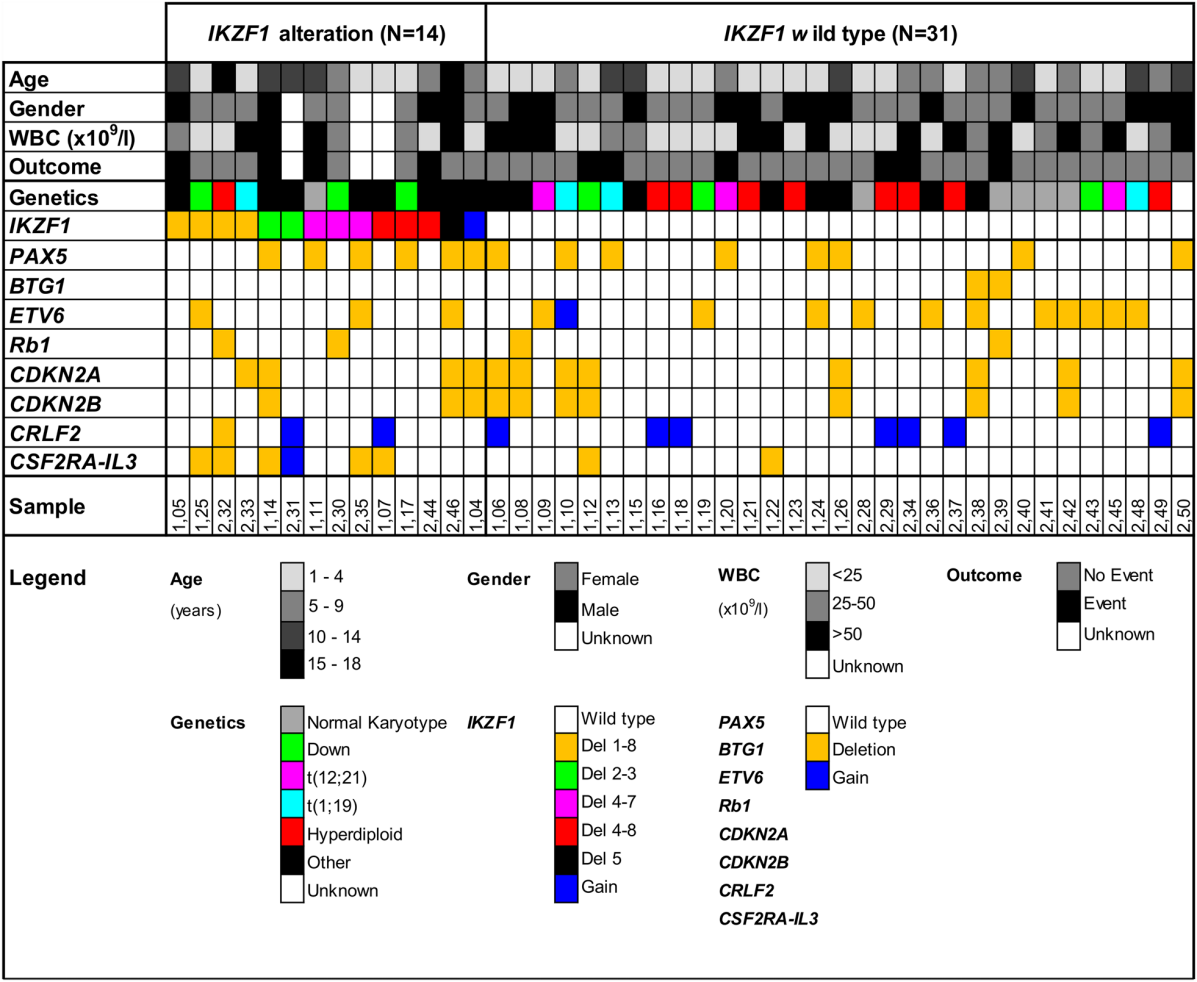
Patients’ characteristics. Clinical and genetic characteristics as well as copy number alterations identified using multiplex ligation-dependent probe amplification (MLPA) analysis are shown for 45 patients who were included in the kinome analysis.

MLPA analysis revealed that among the 109 Philadelphia negative pediatric BCP-ALL patients tested, 17 (15.6%) patients harbor an *IKZF1* deletion whereas one patient (0.9%) showed a gain of *IKZF1*. We generated kinase activity profiles of 31 *IKZF1* wild type patients and 14 patients with an *IKZF1* alteration (13 patients with an *IKZF1* deletion and 1 patient with a gain of *IKZF1*). No material was available for kinome profiling of the other three patients with an *IKZF1* deletion. Among the 14 patients harboring an *IKZF1* alteration, deletions in exons 1 through 8 (4 patients, 28.6%), 4 through 7 (3 patients, 21.4%), and 4 through 8 (3 patients, 21.4%) were most frequent (Fig. 1). Patients’ characteristics are shown in Fig. 1. Patients harboring an *IKZF1* alteration appeared to be older compared to *IKZF1* wild type patients (8.4 versus 5.7 years, respectively, *P* = 0.038). Furthermore, MLPA analysis showed more *CSF2RA* alterations in the group with *IKZF1* alterations compared to the *IKZF1* wild type group (*P* = 0.003).

**Fig. 2:**
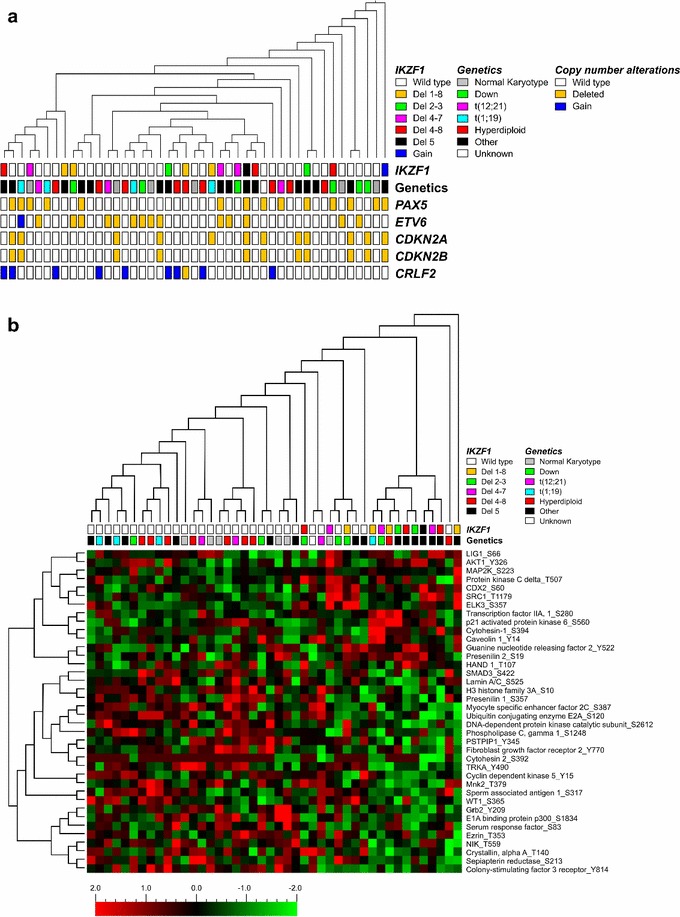
Kinome profile in Philadelphia chromosome negative pediatric BCP-ALL patients. **a** Unsupervised hierarchical clustering of 45 pediatric BCP-ALL cases; 13 *IKZF1* deleted, 1 *IKZF1* gain, and 31 *IKZF1* wild type Philadelphia chromosome negative patients based on 1,008 unique target peptides. No distinct clustering could be observed based on *IKZF1* status, neither on genetic background or other copy number alterations. **b** Unsupervised hierarchical clustering of 44 pediatric BCP-ALL cases; 13 *IKZF1* deleted and 31 *IKZF1* wild type patients based on 38 differentially phosphorylated peptides identified by *t* test.* Each row* represents a peptide,* each column *represents a single ALL sample. The magnitude of deviation from the median is represented by the *color saturation* with each variable normalized to mean 0 and variance 1. *Red* and *green spots* display the phosphorylation intensity above and below the median, respectively.

Kinase activity profiles were generated to reveal potential differences in signaling between *IKZF1* deleted and non-deleted Ph^−^ pediatric BCP-ALL cases. Compared to previously performed kinome arrays, we observed a good variability in peptide phosphorylation intensities among the 1,008 unique target peptides and within the 45 individual patients, indicating that the experimental conditions were optimal. Unsupervised hierarchical cluster analysis showed no clustering based upon *IKZF1* status (Fig. 2a). Moreover, no differential clustering could be observed based on karyotype and copy number alterations of *PAX5*, *ETV6*, *CDKN2A*, *CDKN2B*, and *CRLF2* (Fig. 2a). The clustering in peptide activity was not correlated to patients’ characteristics as age, gender, and white blood cell count (WBC) at diagnosis (Additional file 2: Figure S1). To evaluate whether *IKZF1* deletions display a unique kinase signature we studied differences in peptide phosphorylation intensities by *t*-test analysis. Thirty-eight peptides were differentially phosphorylated between *IKZF1* deleted (*N* = 13) and *IKZF1* wild type (*N* = 31) pediatric Ph^−^ BCP-ALL patients: phosphorylation of 14 peptides was higher in the *IKZF1* deleted group and 24 peptides showed reduced phosphorylation intensities (*P* ≤ 0.05, Fig. 2b, Additional file 3: Table S2). In Fig. 2b, normalized peptide intensities are shown with each variable normalized to mean 0 and variance 1. Although we observed thirty-eight peptides to be differentially phosphorylated, absolute differences of normalized peptide phosphorylation intensities between *IKZF1* deleted and wild type samples were small (Additional file 4: Figure S2; Additional file 3: Table S2). Focusing on the normalized peptide phosphorylation intensities, high phosphorylation levels of peptides derived from Cytohesin-1_S394 and Cytohesin-2_S392 were shown. The list of top 100 most highly phosphorylated peptides also showed only subtle variations between *IKZF1* deleted and wild type cases (Additional file 5: Table S3). Although no gross differences in peptide phosphorylation levels were observed, the list of top 100 highest phosphorylated peptides included a number of potentially druggable targets for the treatment of BCP-ALL. Among the top 100 most highly phosphorylated peptides, we identified 86 peptides previously defined as activated peptides in pediatric ALL including cAMP responsive element binding protein 1 (CREB1), peptides derived from protein kinases related to the PI3K/Akt-signaling pathway including peptides related to phosphatidylinositol 3 kinases and ribosomal S6 kinases, and peptides derived from regulators of the cell cycle including checkpoint kinase 2 and retinoblastoma 1 [19].

### Signal transduction pathway activation in response to *IKZF1* status

**Fig. 3:**
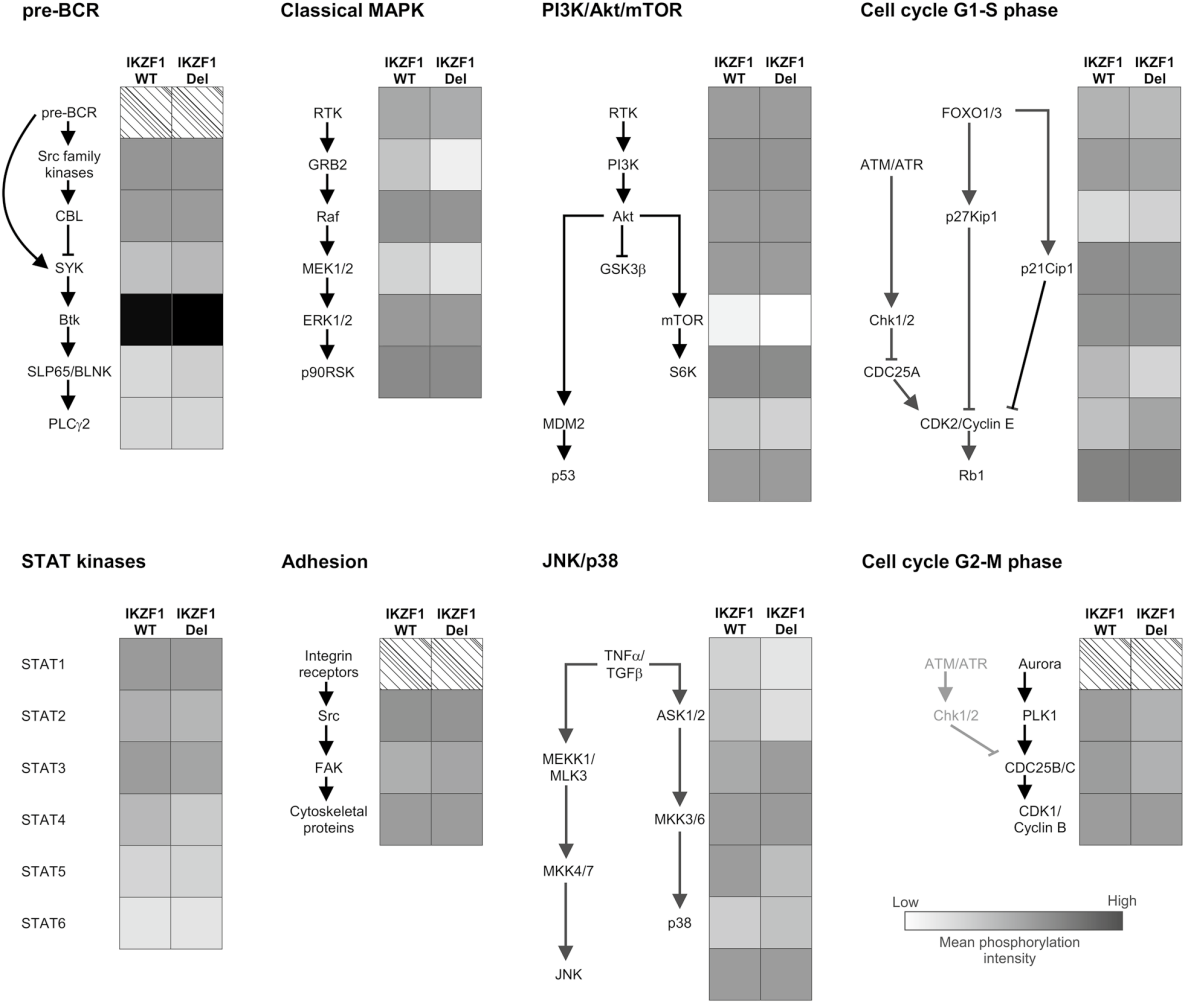
Signal transduction in *IKZF1* deleted versus wild type cases in Philadelphia chromosome negative pediatric BCP-ALL patients. Shown are peptide phosphorylation intensities of proteins involved in important signal transduction pathways for BCP-ALL cell proliferation and survival. Results are expressed in *gray scale* representing the mean peptide phosphorylation intensity of all category peptides.

To focus more closely on active signal transduction pathways, we determined peptide phosphorylation of proteins involved in important signaling pathways for BCP-ALL cell proliferation and survival (e.g. the BCR signaling pathway, the MAPK, PI3K/Akt/mTOR, JAK/STAT5 signaling pathways), adhesion pathways, and regulators of the cell cycle (including p21Cip1 and p27Kip1, Fig. 3). Activity of these signaling pathways could be observed in both *IKZF1* wild type and *IKZF1* deleted pediatric Ph^−^ ALL patients. Comparing phosphorylation intensities of peptides associated with these main signaling pathways showed no differences in pathway activation between *IKZF1* wild type and *IKZF1* deleted Ph^−^ ALL (Additional file 6: Table S4).

### Phosphorylation levels of key signaling proteins in *IKZF1* deleted and wild type BCP-ALL

**Fig. 4:**
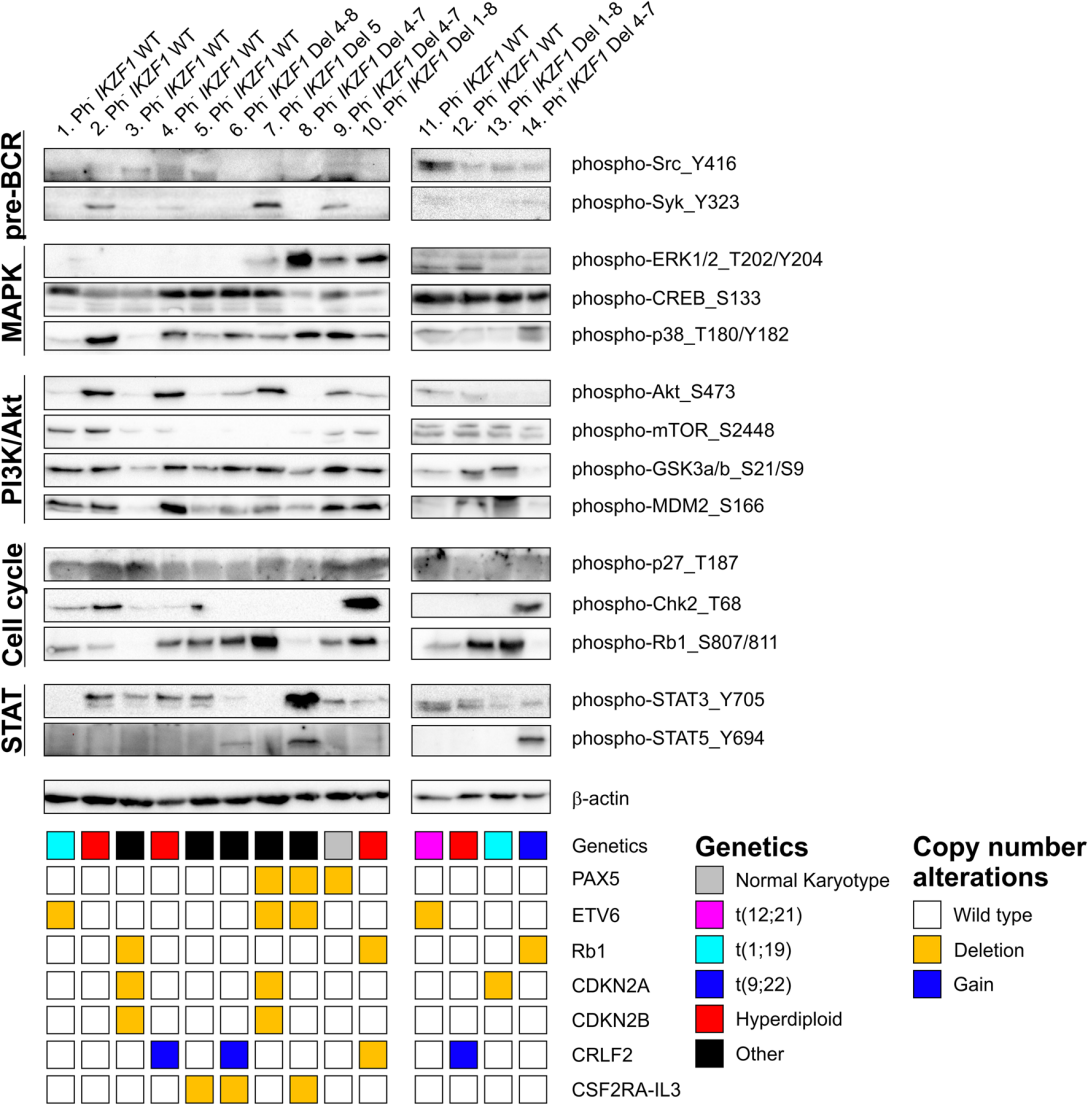
Protein phosphorylation profiles in Philadelphia chromosome negative pediatric BCP-ALL patients. Western blot analysis of 14 pediatric BCP-ALL samples; seven Philadelphia negative *IKZF1* wild type, six Philadelphia negative *IKZF1* deleted patients, and one Philadelphia positive *IKZF1* deleted patient showing phosphorylation expression levels of a selection of proteins. The results show a great diversity in phosphorylation levels between the different ALL samples independently of *IKZF1* status and other copy number alterations.

Previously, upregulation of the B-cell receptor (BCR) signaling pathway was reported in adult Ph^+^ ALL in response to Ikaros-reconstitution, as well as an increased STAT5 phosphorylation in *IKZF1* deleted versus wild type adult ALL cases with unknown cytogenetic background [20, 22]. Although various peptides involved in the BCR signaling pathway and peptides derived from STAT5 were present on the array, we did not observe these differences in our Ph^−^ cases. Therefore, in addition to the kinome profiles, we performed western blot analysis of 14 pediatric BCP-ALL samples; seven Philadelphia negative *IKZF1* wild type patients, six Philadelphia negative *IKZF1* deleted patients, and one Philadelphia positive *IKZF1* deleted patient (Fig. 4). The western blot results showed a large variation in phosphorylation levels between the different ALL samples. STAT5_Y694 phosphorylation could be detected in the Philadelphia positive patient (patient no 14) and in two Philadelphia negative patients with an *IKZF1* deletion (patients no 6 and 8), but was not detectable in the other *IKZF1* deleted Philadelphia negative patients (patients no 7, 9, 10, and 13, Fig. 4). The western blot results showed that protein phosphorylation of Src_Y416, Syk_Y323, ERK1/2_T202/Y204, CREB_S133, p38_T180/Y182, Akt_S473, mTOR_S2448, GSK3α/β_S21/S9, MDM2_S166, p27_T187, Chk2_T68, Rb1_S807/811, and STAT3_Y705 was clearly independent of *IKZF1* status. Neither did we observe a clear relation between the western blot results and known copy number alterations of *PAX5*, *ETV6*, *Rb1*, *CDKN2A*, *CDKN2B*, or *CRLF2* (Fig. 4). In conclusion, our kinome and western blot results suggest that *IKZF1* deletions do not predict a unique protein expression signature in pediatric Ph^−^ ALL.

## Discussion

*IKZF1* deletions are found in approximately 70% of the children with Philadelphia chromosome positive (Ph^+^) ALL and in 10–15% of the children with Philadelphia chromosome negative (Ph^−^) ALL. In both groups, *IKZF1* deletions are associated with an increased risk on relapse and decreased overall survival [2–4]. In Ph^−^ ALL, the effect of *IKZF1* deletions on outcome is most pronounced in children with an intermediate treatment response based on the minimal residual disease at days 42 and 84 [24]. Although multiple studies have established *IKZF1* as a prognostic factor in pediatric BCP-ALL, the effect of *IKZF1* deletions on signaling pathways in Philadelphia negative B-cell precursor ALL is poorly understood. In previous studies, we have shown that kinome profiling can be used successfully to describe active signal transduction pathways in pediatric malignancies [16–19]. In this study we used kinome profiling to elucidate the effect of *IKZF1* deletions on active signal transduction pathways.

Unexpectedly, unsupervised hierarchical cluster analysis revealed no clustering between *IKZF1* deleted and wild type patients. Furthermore, peptide phosphorylation intensities between *IKZF1* deleted and wild type Ph^−^ BCP-ALL patients were very comparable as shown by the phosphorylation intensities of the thirty-eight differentially phosphorylated peptides and by the list of top 100 most highly phosphorylated peptides. While focusing on important signaling pathways involved in cell proliferation and survival of BCP-ALL cells we showed activity of all these pathways, however, no differences between the *IKZF1* deleted versus the *IKZF1* wild type group could be observed. Moreover, western blots of several key proteins involved in ALL signaling showed a variety of phosphorylation events, clearly unrelated to *IKZF1* status.

Although no clear differences in peptide phosphorylation intensities could be observed, kinome profiles showed a remarkable high phosphorylation of peptides derived from Cytohesin-1_S394 and Cytohesin-2_S392. Cytohesins have been described as ErbB receptor activators [25]. It has been described that Cytohesin overexpression enhances epidermal growth factor receptor (EGFR) signaling in human cancers including lung cancer and colorectal cancer [25, 26]. Although kinase domain mutations of ErbB receptors are uncommon in acute leukemias, in vitro inhibition of ErbB2 reduces cell proliferation especially when combined with BCR-ABL tyrosine kinase inhibitors in Ph^+^ ALL, suggesting a role for ErbB signaling pathway activation in leukemia [27, 28]. Therefore, it will be interesting to further explore the role of Cytohesin in BCP-ALL.

Although we did not identify a unique kinome signature as a result of *IKZF1* status, effects of *IKZF1* deletions on gene expression were described previously. Iacobucci et al. showed that *IKZF1* deletions display a unique gene expression signature in a cohort of adult B-ALL patients, including patients with a Philadelphia translocation and B-ALL patients negative for known molecular rearrangements [20]. The gene expression signature was characterized by the downregulation of genes regulating B-cell lineage development and DNA repair upon DNA damage response genes and upregulation of cell cycle/apoptosis genes, JAK/STAT signaling and stem cell self-renewal [20]. More recently, besides an upregulation of genes associated with B-cell proliferation, an upregulation of genes involved in cell adhesion and communication was also observed in pediatric Ph^−^ ALL [29].

In addition to a unique *IKZF1* dependent gene expression profile, a subtype of precursor BCP-ALL with a similar gene expression profile compared to Philadelphia-positive ALL was identified several years ago (Ph-like ALL) [2, 30]. Importantly, 68% of the patients with Ph-like ALL showed deletions in *IKZF1* [31]. Within the Ph-like ALL subtype, 5-year event-free survival rates in patients with *IKZF1* alterations were inferior compared to Ph-like *IKZF1* wild type ALL patients [31]. Recently, Roberts et al. defined the genomic landscape of Ph-like ALL in a large cohort of children and adolescents to elucidate kinase-activating genetic alterations which might include potential leads for targeted therapy [31]. Genomic alterations activating kinase signaling were identified in 91% of the Ph-like ALL patients (*N* = 156) including ABL-class fusions, rearrangements of *JAK2* or *CRLF2*, genetic alterations including *IL7R* and *FLT3*, and Ras pathway mutations [31]. Patients harboring *IKZF1* alterations (*N* = 96, 61.5%) were distributed over all groups of different kinase alterations, indicating a high degree of heterogeneity within the group of *IKZF1* deleted patients [31]. Our results and the results of Roberts et al. suggest that patients harboring *IKZF1* alterations represent a heterogeneous subgroup when evaluated at the level of active signal transduction pathways. The identification of potential targets for tyrosine kinase inhibitors therefore appears to be dictated by upstream genomic alterations that activate kinase signaling or cytokine receptor pathways rather than *IKZF1* status per se.

## Conclusions

The aim of this study was to elucidate the effect of *IKZF1* deletions on active signal transduction pathways using kinome profiling and western blot analysis in children with Ph^−^ BCP-ALL. Although *IKZF1* deletions are an important clinical prognostic factor we were unable to identify a unique *IKZF1* associated protein expression signature in pediatric Ph^−^ BCP-ALL and consequently no specific targets for future therapy of Ph^−^*IKZF1* deleted BCP-ALL were identified.

## Additional files

Additional file 1:
**Table S1.** Data file containing processed raw data values of the PepChip. Additional file 2:
**Figure S1.** Supplementary unsupervised hierarchical clustering. Kinase activity profiles of 45 pediatric BCP-ALL patients were generated. Unsupervised hierarchical clustering of 1,008 unique target peptides using Qlucore Omics Explorer 3.0 showed no distinct clustering based on the patients’ characteristics age, gender and white blood cell count (WBC). Additional file 3:
**Table S2.** List of 38 differentially phosphorylated peptides. Thirty-eight peptides were differentially phosphorylated between *IKZF1* deleted (*N* = 13) and *IKZF1* wild type (*N* = 31) Philadelphia negative pediatric BCP-ALL patients as determined by *t*-test analysis. The phosphorylation of 14 peptides was higher in the *IKZF1* deleted group and 24 peptides showed reduced phosphorylation intensities. Shown are the normalized peptide phosphorylation intensities as well as *P* values. Additional file 4:
**Figure S2.** Absolute phosphorylation intensities of 38 differentially phosphorylated peptides. Supervised hierarchical clustering of 44 pediatric BCP-ALL cases; 13 *IKZF1* deleted and 31 *IKZF1* wild type Philadelphia chromosome negative patients based on 38 peptides identified by t-test. Each row represents a peptide, each column represents a single ALL sample. Absolute phosphorylation intensities are shown by the color saturation, red and green spots display high and low phosphorylation intensities, respectively. Additional file 5:
**Table S3.** Top 100 most highly phosphorylated peptides. Shown is the list of top 100 highest phosphorylated peptides in *IKZF1* deleted and *IKZF1* wild type Philadelphia negative pediatric BCP-ALL patients. Highlighted peptides are overlapping peptides between *IKZF1* deleted and *IKZF1* wild type. Additional file 6:
**Table S4.** List of proteins involved in important signaling pathways for BCP-ALL. Shown are proteins involved in important signaling pathways for BCP-ALL cell proliferation and survival (e.g. the BCR signaling pathway, the MAPK, PI3 K/Akt/mTOR, JAK/STAT5 signaling pathways), adhesion pathways, and regulators of the cell cycle (including p21Cip1 and p27Kip1). The mean normalized phosphorylation intensities of multiple peptides derived from indicated proteins as well as *P*-values are shown for *IKZF1* deleted (*N* = 13) and *IKZF1* wild type (*N* = 31) pediatric patients.

## Abbreviations

ALL: acute lymphoblastic leukemia
BCP-ALL: B-cell progenitor acute lymphoblastic leukemia
BCR: B-cell receptor
BTK: Bruton’s tyrosine kinase
CK2: casein kinase II
MLPA: multiplex ligation-dependent probe amplification
Ph^−^: Philadelphia chromosome negative
Ph^+^: Philadelphia chromosome positive
SYK: spleen tyrosine kinase

## References

[1] Inaba H, Greaves M, Mullighan CG (2013). Acute lymphoblastic leukaemia. Lancet.

[2] Mullighan CG, Su X, Zhang J, Radtke I, Phillips LA, Miller CB (2009). Deletion of IKZF1 and prognosis in acute lymphoblastic leukemia. N Engl J Med.

[3] Kuiper RP, Waanders E, van der Velden VH, van Reijmersdal SV, Venkatachalam R, Scheijen B (2010). IKZF1 deletions predict relapse in uniformly treated pediatric precursor B-ALL. Leukemia.

[4] van der Veer A, Waanders E, Pieters R, Willemse ME, Van Reijmersdal SV, Russell LJ (2013). Independent prognostic value of BCR-ABL1-like signature and IKZF1 deletion, but not high CRLF2 expression, in children with B-cell precursor ALL. Blood.

[5] Mullighan CG, Zhang J, Harvey RC, Collins-Underwood JR, Schulman BA, Phillips LA (2009). JAK mutations in high-risk childhood acute lymphoblastic leukemia. Proc Natl Acad Sci USA.

[6] Olsson L, Albitar F, Castor A, Behrendtz M, Biloglav A, Paulsson K (2015). Cooperative genetic changes in pediatric B-cell precursor acute lymphoblastic leukemia with deletions or mutations of IKZF1. Genes Chromosom Cancer.

[7] Olsson L, Johansson B (2015). Ikaros and leukaemia. Br J Haematol.

[8] Georgopoulos K, Bigby M, Wang JH, Molnar A, Wu P, Winandy S (1994). The Ikaros gene is required for the development of all lymphoid lineages. Cell.

[9] Wang JH, Nichogiannopoulou A, Wu L, Sun L, Sharpe AH, Bigby M (1996). Selective defects in the development of the fetal and adult lymphoid system in mice with an Ikaros null mutation. Immunity.

[10] Winandy S, Wu P, Georgopoulos K (1995). A dominant mutation in the Ikaros gene leads to rapid development of leukemia and lymphoma. Cell.

[11] Gomez-del Arco P, Maki K, Georgopoulos K (2004). Phosphorylation controls Ikaros’s ability to negatively regulate the G(1)-S transition. Mol Cell Biol..

[12] Uckun FM, Ma H, Zhang J, Ozer Z, Dovat S, Mao C (2012). Serine phosphorylation by SYK is critical for nuclear localization and transcription factor function of Ikaros. Proc Natl Acad Sci USA.

[13] Ma H, Qazi S, Ozer Z, Zhang J, Ishkhanian R, Uckun FM (2013). Regulatory phosphorylation of Ikaros by Bruton’s tyrosine kinase. PLoS One.

[14] Dupuis A, Gaub MP, Legrain M, Drenou B, Mauvieux L, Lutz P (2013). Biclonal and biallelic deletions occur in 20% of B-ALL cases with IKZF1 mutations. Leukemia.

[15] Sun L, Liu A, Georgopoulos K (1996). Zinc finger-mediated protein interactions modulate Ikaros activity, a molecular control of lymphocyte development. EMBO J.

[16] Sikkema AH, Diks SH, den Dunnen WF, ter Elst A, Scherpen FJ, Hoving EW (2009). Kinome profiling in pediatric brain tumors as a new approach for target discovery. Cancer Res.

[17] Ter Elst A, Diks SH, Kampen KR, Hoogerbrugge PM, Ruijtenbeek R, Boender PJ (2011). Identification of new possible targets for leukemia treatment by kinase activity profiling. Leuk Lymphoma.

[18] Kampen KR, Ter Elst A, Mahmud H, Scherpen FJ, Diks SH, Peppelenbosch MP (2014). Insights in dynamic kinome reprogramming as a consequence of MEK inhibition in MLL-rearranged AML. Leukemia.

[19] van der Sligte NE, Scherpen FJ, Meeuwsen-de Boer TG, Lourens HJ, Ter Elst A, Diks SH (2015). Kinase activity profiling reveals active signal transduction pathways in pediatric acute lymphoblastic leukemia: a new approach for target discovery. Proteomics.

[20] Iacobucci I, Iraci N, Messina M, Lonetti A, Chiaretti S, Valli E (2012). IKAROS deletions dictate a unique gene expression signature in patients with adult B-cell acute lymphoblastic leukemia. PLoS One.

[21] Safavi S, Hansson M, Karlsson K, Biloglav A, Johansson B, Paulsson K (2015). Novel gene targets detected by genomic profiling in a consecutive series of 126 adults with acute lymphoblastic leukemia. Haematologica.

[22] Trageser D, Iacobucci I, Nahar R, Duy C, von Levetzow G, Klemm L (2009). Pre-B cell receptor-mediated cell cycle arrest in Philadelphia chromosome-positive acute lymphoblastic leukemia requires IKAROS function. J Exp Med.

[23] Dokter WH, Tuyt L, Sierdsema SJ, Esselink MT, Vellenga E (1995). The spontaneous expression of interleukin-1 beta and interleukin-6 is associated with spontaneous expression of AP-1 and NF-kappa B transcription factor in acute myeloblastic leukemia cells. Leukemia.

[24] Waanders E, van der Velden VH, van der Schoot CE, van Leeuwen FN, van Reijmersdal SV, de Haas V (2011). Integrated use of minimal residual disease classification and IKZF1 alteration status accurately predicts 79% of relapses in pediatric acute lymphoblastic leukemia. Leukemia.

[25] Bill A, Schmitz A, Albertoni B, Song JN, Heukamp LC, Walrafen D (2010). Cytohesins are cytoplasmic ErbB receptor activators. Cell.

[26] Pan T, Sun J, Hu J, Hu Y, Zhou J, Chen Z (2014). Cytohesins/ARNO: the function in colorectal cancer cells. PLoS One.

[27] Irwin ME, Nelson LD, Santiago-O’Farrill JM, Knouse PD, Miller CP, Palla SL (2013). Small molecule ErbB inhibitors decrease proliferative signaling and promote apoptosis in philadelphia chromosome-positive acute lymphoblastic leukemia. PLoS One.

[28] Lee JW, Soung YH, Kim SY, Nam SW, Park WS, Lee JY (2006). Kinase domain mutation of ERBB family genes is uncommon in acute leukemias. Leuk Res.

[29] Vitanza NA, Zaky W, Blum R, Meyer JA, Wang J, Bhatla T (2014). Ikaros deletions in BCR-ABL-negative childhood acute lymphoblastic leukemia are associated with a distinct gene expression signature but do not result in intrinsic chemoresistance. Pediatr Blood Cancer.

[30] Den Boer ML, van Slegtenhorst M, De Menezes RX, Cheok MH, Buijs-Gladdines JG, Peters ST (2009). A subtype of childhood acute lymphoblastic leukaemia with poor treatment outcome: a genome-wide classification study. Lancet Oncol..

[31] Roberts KG, Li Y, Payne-Turner D, Harvey RC, Yang YL, Pei D (2014). Targetable kinase-activating lesions in Ph-like acute lymphoblastic leukemia. N Engl J Med.

