# Supplementary material for: Effect of IKZF1 deletions on signal transduction pathways in Philadelphia chromosome negative pediatric B-cell precursor acute lymphoblastic leukemia (BCP-ALL)
Source: Exp Hematol Oncol. 2015 Aug 12;4:23. doi: 10.1186/s40164-015-0017-y (PMC4534008; doi:10.1186/s40164-015-0017-y)

Supplementary Figure 1. Supplementary unsupervised hierarchical clustering

Kinase activity profiles of 45 pediatric BCP-ALL patients were generated. Unsupervised hierarchical clustering of 1,008 unique target peptides using Qlucore Omics Explorer 3.0 showed no distinct clustering based on the patients' characteristics age, gender and white blood cell count (WBC).

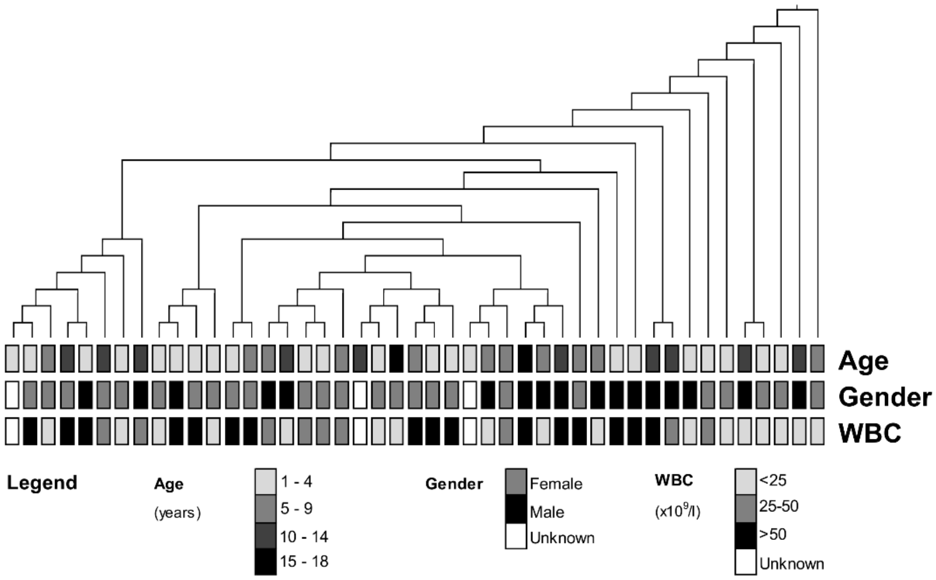

Supplement: Additional file 2: — Figure S1. Supplementary unsupervised hierarchical clustering. Kinase activity profiles of 45 pediatric BCP-ALL patients were generated. Unsupervised hierarchical clustering of 1,008 unique target peptides using Qlucore Omics Explorer 3.0 showed no distinct clustering based on the patients’ characteristics age, gender and white blood cell count (WBC). [file 40164_2015_17_MOESM2_ESM.pdf]
