# Supplementary material for: Effect of IKZF1 deletions on signal transduction pathways in Philadelphia chromosome negative pediatric B-cell precursor acute lymphoblastic leukemia (BCP-ALL)
Source: Exp Hematol Oncol. 2015 Aug 12;4:23. doi: 10.1186/s40164-015-0017-y (PMC4534008; doi:10.1186/s40164-015-0017-y)
Supplement: Additional file 3: — Table S2. List of 38 differentially phosphorylated peptides. Thirty-eight peptides were differentially phosphorylated between IKZF1 deleted (N = 13) and IKZF1 wild type (N = 31) Philadelphia negative pediatric BCP-ALL patients as determined by t-test analysis. The phosphorylation of 14 peptides was higher in the IKZF1 deleted group and 24 peptides showed reduced phosphorylation intensities. Shown are the normalized peptide phosphorylation intensities as well as P values. [file 40164_2015_17_MOESM3_ESM.pdf]

Supplementary Table 2. List of 38 differentially phosphorylated peptides

Thirty-eight peptides were differentially phosphorylated between IKZF1 deleted (*N* = 13) and IKZF1 wild type (*N* = 31) pediatric Ph- BCP-ALL patients as determined by *t*-test analysis. The phosphorylation of 14 peptides was higher in the IKZF1 deleted group and 24 peptides showed reduced phosphorylation. Shown are the mean normalized peptide phosphorylation intensities as well as the *P*-value.

**Peptide**	**Mean normalized phosphorylation intensity *IKZF1* wild type**	**Mean normalized phosphorylation intensity *IKZF1* deleted**	***P*-value**
LIG1_S66	-16.05	85.92	0.027
AKT1_Y326	-61.45	24.09	0.017
MAP2K3_S223	57.79	260.95	0.046
Protein kinase C delta_T507	-32.28	80.31	0.031
CDX2_S60	159.80	271.69	0.029
SRC1_T1179	19.30	160.26	0.010
ELK3_S357	126.12	331.38	0.015
Transcription factor IIA, 1_S280	103.29	287.26	0.006
p21 activated protein kinase 6_S560	125.98	281.18	0.008
Cytohesin-1_S394	19105.75	22027.02	0.003
Caveolin 1_Y14	5.73	109.67	0.026
Guanine nucleotide releasing factor 2_Y522	0.16	78.66	0.027
Presenilin 2_S19	-64.82	28.79	0.030
HAND1_T107	-24.53	73.40	0.024
SMAD3_S422	-41.20	-136.92	0.013
Lamin A/C_S525	35.68	-39.31	0.031
H3 histone, family 3A_S10	11.01	-83.46	0.045
Presenilin 1_S357	30.04	-70.42	0.008
Myocyte specific enhancer factor 2C_S387	61.79	-85.96	0.005
Ubiquitin conjugating enzyme E2A_S120	24.47	-81.90	0.003
DNA-dependent protein kinase catalytic subunit_S2612	32.60	-46.41	0.045
Phospholipase C, gamma1_S1248	142.43	9.38	0.024
PSTPIP1_Y345	15.59	-88.69	0.002
Fibroblast growth factor receptor 2_Y770	25.30	-71.89	0.014
Cytohesin 2_S392	27404.69	23547.16	0.005
TRKA_Y490	8.24	-89.56	0.002
Cyclin dependent kinase 5_Y15	117.92	-0.13	0.021
Mnk2_T379	45.11	-56.85	0.009
Sperm associated antigen 1_S317	147.22	55.48	0.039
WT1_S365	-6.55	-83.01	0.049
Grb2_Y209	11.25	-88.94	0.020
E1A binding protein p300_S1834	-11.51	-116.11	0.007
Serum response factor_S83	92.38	4.36	0.021
Ezrin_T353	44.67	-55.24	0.019
NIK_T559	18.84	-58.15	0.034
Crystallin, alpha A_T140	3.64	-82.50	0.048
Sepiapterin reductase_S213	59.56	-37.92	0.012
Colony-stimulating factor 3 receptor_Y814	-3.18	-87.53	0.006
