# Supplementary material for: Effect of IKZF1 deletions on signal transduction pathways in Philadelphia chromosome negative pediatric B-cell precursor acute lymphoblastic leukemia (BCP-ALL)
Source: Exp Hematol Oncol. 2015 Aug 12;4:23. doi: 10.1186/s40164-015-0017-y (PMC4534008; doi:10.1186/s40164-015-0017-y)

Supplementary Figure 2 Absolute phosphorylation intensities of 38 differentially phosphorylated peptides

Supervised hierarchical clustering of 44 pediatric BCP-ALL cases; 13 *IKZF1* deleted and 31 *IKZF1* wild type Philadelphia chromosome negative patients based on 38 peptides identified by *t*-test. Each row represents a peptide, each column represents a single ALL sample. Absolute phosphorylation intensities are shown by the color saturation, red and green spots display high and low phosphorylation intensities, respectively.

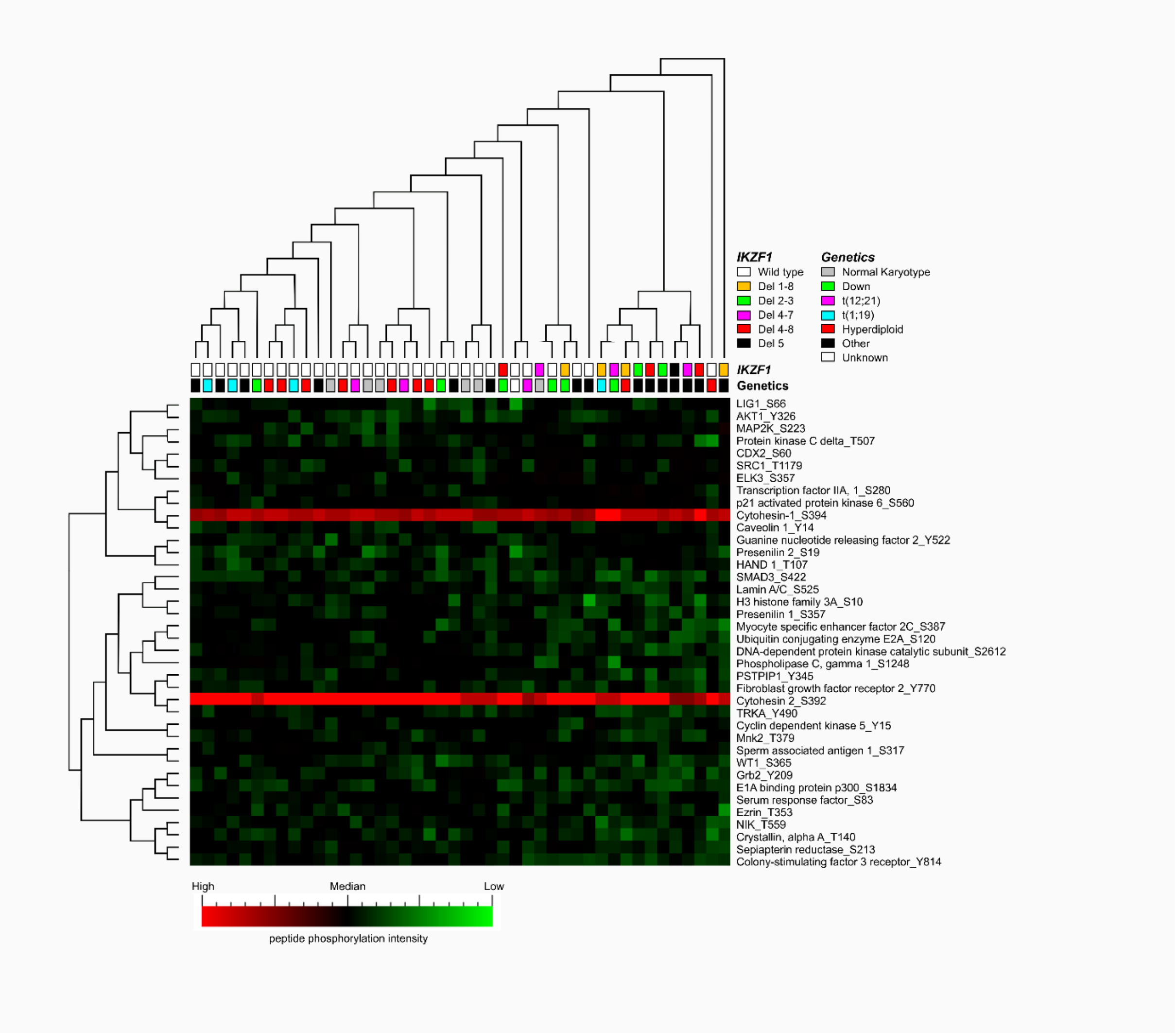

Supplement: Additional file 4: — Figure S2. Absolute phosphorylation intensities of 38 differentially phosphorylated peptides. Supervised hierarchical clustering of 44 pediatric BCP-ALL cases; 13 IKZF1 deleted and 31 IKZF1 wild type Philadelphia chromosome negative patients based on 38 peptides identified by t-test. Each row represents a peptide, each column represents a single ALL sample. Absolute phosphorylation intensities are shown by the color saturation, red and green spots display high and low phosphorylation intensities, respectively. [file 40164_2015_17_MOESM4_ESM.pdf]
