# Supplementary material for: Effect of IKZF1 deletions on signal transduction pathways in Philadelphia chromosome negative pediatric B-cell precursor acute lymphoblastic leukemia (BCP-ALL)
Source: Exp Hematol Oncol. 2015 Aug 12;4:23. doi: 10.1186/s40164-015-0017-y (PMC4534008; doi:10.1186/s40164-015-0017-y)
Supplement: Additional file 5: — Table S3. Top 100 most highly phosphorylated peptides. Shown is the list of top 100 highest phosphorylated peptides in IKZF1 deleted and IKZF1 wild type Philadelphia negative pediatric BCP-ALL patients. Highlighted peptides are overlapping peptides between IKZF1 deleted and IKZF1 wild type. [file 40164_2015_17_MOESM5_ESM.pdf]

Supplementary Table 2. Top 100 most highly phosphorylated peptides

Highlighted peptides are overlapping peptides between *IKZF1* deleted and *IKZF1* wild type.

***IKZF1* deleted**	***IKZF 1* Wild type**
Cytohesin 2_S392	Cytohesin 2_S392
Cytohesin-1_S394	Cytohesin-1_S394
Metabotropic glutamate receptor 1_T695	Metabotropic glutamate receptor 1_T695
Coilin_S184	Coilin_S184
APC_S2054	APC_S2054
PEA15_S104	PEA15_S104
Bruton's tyrosine kinase_S180	Bruton's tyrosine kinase_S180
RAP1 GTPase activating protein 1_S484	RAP1 GTPase activating protein 1_S484
Small nuclear ribonucleoprotein 70 kD_S137	Small nuclear ribonucleoprotein 70 kD_S137
Splicing factor 1_S20	Neurogranin_S36
Syntaxin binding protein 1_S313	Splicing factor 1_S20
Neurogranin_S36	Syntaxin binding protein 1_S313
Retinoblastoma like 1_S964	Guanine nucleotide binding protein, alpha 15 subunit_S336
Retinoblastoma like 2_S1035	Retinoblastoma like 1_S964
Guanine nucleotide binding protein, alpha 15 subunit_S336	cAMP response element-binding protein 1_S133
Ras related protein 1A_S180	Ras related protein 1A_S180
Beta-2-adrenergic receptor_S262	CDC6_S54
CDC6_S54	Beta-2-adrenergic receptor_S262
Glutamate receptor ionotropic, AMPA 4_S862	Ras related protein Rap 1B_S179
cAMP response element-binding protein 1_S133	Retinoblastoma like 2_S1035
Ras related protein Rap 1B_S179	CCAAT/Enhancer binding protein, beta_T235
CCAAT/Enhancer binding protein, beta_T235	CNPase_S9
Peroxisome proliferator activated receptor, gamma_S112	RhoA_S188
Vitronectin_S381	Vitronectin_S381
HMG14_S7	Glutamate receptor ionotropic, AMPA 4_S862
CNPase_S9	Peroxisome proliferator activated receptor, gamma_S112
Desmin_S12	HMG14_S7
RhoA_S188	Kinetochore associated 2_S165
HLA-A_S337	Desmin_S12
14-3-3-Eta_S59;S60	HLA-A_S337
Retinoblastoma 1_S807	Retinoblastoma 1_S807
6-phosphofructo-2-kinase/fructose-2,6-biphosphatase 2_S483	14-3-3-Eta_S59;S60
Regulator of G protein signaling 10_S176	Formyl peptide receptor-like 1_S236
Kinetochore associated 2_S165	Regulator of G protein signaling 10_S176
Formyl peptide receptor-like 1_S236	6-phosphofructo-2-kinase/fructose-2,6-biphosphatase 2_S483
High mobility group AT-hook 2_S59	Peptidylglycine alpha amidating monooxygenase_S930
Peptidylglycine alpha amidating monooxygenase_S930	eIF2 alpha_S52
Ras associated protein Rab4_S204	Ryanodine receptor 2_S2808
eIF2 alpha_S52	Ribosomal S6 kinase 1_S230

CHK2_S19	HMG17_S25
HMG17_S25	LKB1_S424
c-Myc_T58	cAMP-specific 3',5'-cyclic phosphodiesterase 4B_S133
Ryanodine receptor 2_S2808	Ras associated protein Rab4_S204
Opioid receptor mu 1_S268	Transcription elongation factor A- like1_S37
cAMP-specific 3',5'-cyclic phosphodiesterase 4B_S133	ATP2B1_T1117
Ribosomal S6 kinase 1_S230	c-Myc_T58
Transcription elongation factor A- like1_S37	High mobility group AT-hook 2_S59
LKB1_S424	Synapsin II_S10
Activating transcription factor 1_S63	ADAM 12_Y907
ATP2B1_T1117	CHK2_S19
Neutrophil cytosolic factor 4 40kDa_T154	c-Src_S12
ADAM 12_Y907	Opioid receptor mu 1_S268
Fascin 1_S38	Activating transcription factor 1_S63
ELK 1_S383	Tuberin_S939
Tuberin_S939	Neutrophil cytosolic factor 4 40kDa_T154
c-Src_S12	SHP2_S591
SHP2_S591	ELK 1_S383
PKR_S242	PKR_S242
Synapsin II_S10	RAF1_S259
SHP2_Y542	Heterogeneous nuclear ribonucleoprotein K_S302
Connexin 32_S233	Fascin 1_S38
Protein phosphatase 2, regulatory subunit B56, alpha_S28	Connexin 32_S233
Ribonucleotide reductase M2 subunit_S20	Ribonucleotide reductase M2 subunit_S20
RAF1_S259	Complement component 5 receptor 1_S314
RAD9_S336	Retinoblastoma like 1_S975
Retinoblastoma like 1_S975	Protein phosphatase 2, regulatory subunit B56, alpha_S28
ATP2B1_S1178	ATP2B1_S1178
Keratin 8_S74	Keratin 8_S74
SPIB transcription factor_T56	Flap endonuclease-1_S187
Glycogen synthase 1_S645	RAD9_S336
Retinoblastoma 1_S795	SHP2_Y542
Heterogeneous nuclear ribonucleoprotein K_S302	Glycogen synthase 1_S645
Complement component 5 receptor 1_S314	Guanine nucleotide binding protein, alpha Z polypeptide_S27
NUP210_S1881	Retinoblastoma 1_S795
ELK3_S363	Connexin 43_S367
Alpha 2A adrenergic receptor_S232	BRCA1_S509
Keratin 8_S431	Keratin 8_S431
SMAD2_T220	Ras related C3 botulinum toxin substrate 1_S71
Lamin B1_S395	c-Myc_S62
NPRA_S538	Wiskott-Aldrich syndrome protein interacting protein_S488
Guanine nucleotide binding protein, alpha Z polypeptide_S27	SPIB transcription factor_T56
c-Myc_S62	Nucleolar phosphoprotein p130_S623
Connexin 43_S367	Lamin B1_S395
p53_S33	NIPP1_S199
Heat-shock transcription factor 1_S303	Alpha 2A adrenergic receptor_S232

Phosphatidylinositol 3 kinase, regulatory subunit, alpha_Y580	ELK3_S363
B-Myb_S577	NUP210_S1881
Lamin B1_S23	Protein phosphatase 1, regulatory subunit 3A_S48
Lamin A/C_S22	B-Myb_S577
Dematin_S403	SMAD2_T220
LIM domain kinase 1_T505	Ribosomal protein S6 kinase alpha 4_S347
Nucleolar phosphoprotein p130_S623	Lamin B1_S23
Ras related C3 botulinum toxin substrate 1_S71	Aquaporin 0_S331
Kell blood group protein_S63	p53_S33
TCFL1_S41	CALDESMON 1_S759
Aquaporin 0_S331	BCL2 antagonist of cell death_S75
CALDESMON 1_S759	LIM domain kinase 1_T505
Ribosomal protein S6 kinase alpha 4_S347	Phosphatidylinositol 3 kinase, regulatory subunit, alpha_Y580
Synapsin I_S551	Kell blood group protein_S63
Down syndrome critical region protein 1_S112	Cyclin-dependent kinase inhibitor 1A_S146
