# Supplementary material for: Effect of IKZF1 deletions on signal transduction pathways in Philadelphia chromosome negative pediatric B-cell precursor acute lymphoblastic leukemia (BCP-ALL)
Source: Exp Hematol Oncol. 2015 Aug 12;4:23. doi: 10.1186/s40164-015-0017-y (PMC4534008; doi:10.1186/s40164-015-0017-y)
Supplement: Additional file 6: — Table S4. List of proteins involved in important signaling pathways for BCP-ALL. Shown are proteins involved in important signaling pathways for BCP-ALL cell proliferation and survival (e.g. the BCR signaling pathway, the MAPK, PI3 K/Akt/mTOR, JAK/STAT5 signaling pathways), adhesion pathways, and regulators of the cell cycle (including p21Cip1 and p27Kip1). The mean normalized phosphorylation intensities of multiple peptides derived from indicated proteins as well as P-values are shown for IKZF1 deleted (N = 13) and IKZF1 wild type (N = 31) pediatric patients. [file 40164_2015_17_MOESM6_ESM.pdf]

Supplementary Table 4. List of proteins involved in important signaling pathways for BCP-ALL

Shown are proteins involved in important signaling pathways for BCP-ALL cell proliferation and survival (e.g. the BCR signaling pathway, the MAPK, PI3K/Akt/mTOR, JAK/STAT5 signaling pathways), adhesion pathways, and regulators of the cell cycle (including p21Cip1 and p27Kip1). The mean normalized phosphorylation intensities of multiple peptides derived from indicated proteins as well as *P*-values are shown for *IKZF1* deleted (*N* = 13) and *IKZF1* wild type (*N* = 31) pediatric patients.

pre-BCR pathway

**Protein**	**Mean normalized phosphorylation intensity *IKZF1* wild type**	**Mean normalized phosphorylation intensity *IKZF1* deleted**	***P*-value**
Src family kinases	418.39	371.61	0.456
CBL	145.17	116.62	0.608
SYK	16.89	28.98	0.651
Btk	5112.98	5146.52	0.902
SLP65/BLNK	-15.33	-1.63	0.675
PLCγ2	-13.69	-12.72	0.975

Classical MAPK pathway

**Protein**	**Mean normalized phosphorylation intensity *IKZF1* wild type**	**Mean normalized phosphorylation intensity *IKZF1* deleted**	***P*-value**
RTK	51.75	46.44	0.696
GRB2	11.25	-50.76	0.197
Raf	553.36	455.05	0.442
MEK1/2	-8.83	-32.17	0.417
ERK1/2	199.14	185.71	0.794
P90RSK	957.09	912.94	0.755

PI3K/Akt/mTOR pathway

**Protein**	**Mean normalized phosphorylation intensity *IKZF1* wild type**	**Mean normalized phosphorylation intensity *IKZF1* deleted**	***P*-value**
RTK	89.63	105.17	0.529
PI3K	439.62	442.33	0.979
Akt	92.90	152.20	0.040
GSK3β	107.25	67.76	0.305
mTOR	-56.54	-75.21	0.442
S6K	948.98	877.50	0.578
MDM2	1.80	-6.06	0.774
p53	134.32	140.80	0.746

Cell cycle G1-S phase

**Protein**	**Mean normalized phosphorylation intensity *IKZF1* wild type**	**Mean normalized phosphorylation intensity *IKZF1* deleted**	***P*-value**
FOXO1/3	37.56	25.44	0.728
ATM/ATR	88.87	58.90	0.483
p27Kip1	-18.63	-6.98	0.579
p21Cip1	741.00	572.50	0.377
Chk1/2	531.19	569.72	0.628
CDC25A	29.76	-10.94	0.108
CDK2/Cyclin E	17.36	55.80	0.357
Rb1	1266.82	1371.70	0.411

STAT kinases

**Protein**	**Mean normalized phosphorylation intensity *IKZF1* wild type**	**Mean normalized phosphorylation intensity *IKZF1* deleted**	***P*-value**
STAT1	174.35	153.28	0.547
STAT2	42.05	32.30	0.831
STAT3	98.53	56.58	0.275
STAT4	29.66	2.37	0.575
STAT5	-10.74	-8.80	0.931
STAT6	-35.02	-34.97	0.999

Adhesion

**Protein**	**Mean normalized phosphorylation intensity *IKZF1* wild type**	**Mean normalized phosphorylation intensity *IKZF1* deleted**	***P*-value**
Src	525.62	457.95	0.385
FAK	42.38	54.65	0.755
Cytoskeletal proteins	93.29	74.16	0.207

JNK/p38 pathway

**Protein**	**Mean normalized phosphorylation intensity *IKZF1* wild type**	**Mean normalized phosphorylation intensity *IKZF1* deleted**	***P*-value**
TNFα/TGFβ	-7.95	-35.81	0.175
ASK1/2	19.97	-24.97	0.490
MEKK1/MLK3	49.42	74.70	0.398
MKK3/6	88.39	168.72	0.151
MKK4/7	75.41	20.16	0.323
p38	-1.12	14.89	0.648
JNK	104.19	78.69	0.621

Cell cycle G2-M phase

**Protein**	**Mean normalized phosphorylation intensity *IKZF1* wild type**	**Mean normalized phosphorylation intensity *IKZF1* deleted**	***P*-value**
PLK1	71.10	38.56	0.541
CDC25B/C	65.73	39.06	0.322
CDK1/Cyclin B	79.97	73.61	0.862
